# Establishment and Validation of a Genetic Label Associated With M2 Macrophage Infiltration to Predict Survival in Patients With Colon Cancer and to Assist in Immunotherapy

**DOI:** 10.3389/fgene.2021.726387

**Published:** 2021-09-06

**Authors:** Boyang Xu, Ziqi Peng, Guanyu Yan, Ningning Wang, Moye Chen, Xue Yao, Mingjun Sun, Yue An

**Affiliations:** ^1^Department of Gastroenterology, The First Affiliated Hospital of China Medical University, Shenyang, China; ^2^Department of Breast Surgery, The First Affiliated Hospital of China Medical University, Shenyang, China; ^3^Department of Surgical Oncology, The First Hospital of China Medical University, Shenyang, China; ^4^Department of Endoscopy, The First Hospital of China Medical University, Shenyang, China

**Keywords:** colon cancer, immunotherapy, infiltration, M2 macrophages, M2 macrophage score

## Abstract

**Background:**

Colon cancer is a malignant tumor with high morbidity and mortality. Researchers have tried to interpret it from different perspectives and divided it into different subtypes to facilitate individualized treatment. With the rise in the use of immunotherapy, its value in the field of tumor has begun to emerge. From the perspective of immune infiltration, this study classified colon cancer according to the infiltration of M2 macrophages in patients with colon cancer and further explored the same.

**Methods:**

Cibersort algorithm was used to analyze the level of immune cell infiltration in patients with colon cancer in The Cancer Genome Atlas (TCGA) database. Weighted gene co-expression network analysis (WGCNA), Consensus Clustering analysis, Lasso analysis, and univariate Kaplan–Meier analysis were used to screen and verify the hub genes associated with M2 macrophages. Principal component analysis (PCA) was used to establish the M2 macrophage-related score (M2I Score). The correlation between M2I Score and somatic cell variation and microsatellite instability (MSI) were analyzed. Furthermore, the correlation between M2 macrophage score and differences in immunotherapy sensitivity was also explored.

**Results:**

M2 macrophage infiltration was associated with poor prognosis. Four hub genes (ANKS4B, CTSD, TIMP1, and ZNF703) were identified as the progression-related genes associated with M2 macrophages. A stable and accurate M2I Score for M2 macrophages used in colon adenocarcinoma was determined based on four hub genes. The M2I Score was positively correlated with the tumor mutation load (TMB). The M2I Score of the group with high instability of microsatellites was higher than that of the group with low instability of microsatellites and microsatellite-stable group. Combined with the Cancer Immunome Atlas database, we concluded that patients with high M2I Scores were more sensitive to programmed cell death protein 1 (PD-1) inhibitors and PD-1 inhibitors combined with cytotoxic T-lymphocyte–associated antigen 4 (CTLA-4) inhibitors. The low-rating group may have better efficacy without immune checkpoint inhibitors or with CTLA4 inhibitors alone.

**Conclusion:**

Four prognostic hub genes associated with M2 macrophages were screened to establish the M2I Score. Patients were divided into two subgroups: high M2I Score group and low M2I Score group. TMB, MSI, and sensitivity to immunotherapy were higher in the high-rated group. PD-1 inhibitors or PD-1 combined with CTLA-4 inhibitors are preferred for patients in the high-rated group who are more sensitive to immunotherapy.

## Introduction

According to the latest global cancer data released by the International Agency for Research on Cancer of the World Health Organization in 2020, colon cancer accounted for 10% of the new cases of all malignant tumors, ranking third among all malignant tumors, with deaths reaching nearly 940,000 cases, accounting for 9.4% of all cancer deaths (Siegel et al., 2020). Despite continued research and improvement in treatment regimens for colon cancer, the 5-year survival rate for patients with colon cancer remains low (Siegel et al., 2014). In recent years, immunotherapy has attracted a great deal of attention for its success in treating previously difficult solid tumors, such as melanoma and lung cancer (Amm et al., 2018; Gandhi et al., 2018). In colon cancer, immunotherapy, particularly immune checkpoint inhibitor therapy, has shown promising results in patients with mismatched repair defects or high levels of microsatellite instability (MSI-H); it was approved by regulatory agencies in 2017 to treat tumors with severe mutations (Ganesh et al., 2019).

Macrophages are one of the most abundant white blood cells in the colon, which play an important role in a variety of intestinal diseases, such as inflammatory bowel disease and bowel cancer (Lee et al., 1985; Platt et al., 2010). Tumor-associated macrophages have different polarization directions, among which M1 macrophages are called pro-inflammatory macrophages, whereas M2 macrophages are called anti-inflammatory macrophages (Mantovani et al., 2004). In tumors, M1 macrophages can effectively clear tumor cells by presenting antigen to T cells, activating specific immune response, regulating and promoting the immune response of T helper (Th)1 cells (Sica and Mantovani, 2012). In contrast, M2 macrophages inhibit the proliferation and activation of T cells by secreting immunosuppressive factors, cytokines, and growth factors, regulate and promote Th2 immune response, promote the growth of tumor cells, participate in tumor angiogenesis, and promote tumor invasion and metastasis (Biswas et al., 2006). Both M1 and M2 macrophages exist in the tumor microenvironment, but with the progress of the tumor, M2 macrophages gradually increase in proportion, leading to a poor prognosis of the patients (Badawi et al., 2015). Because of the importance of macrophages in tumors, tumor therapy strategies targeting macrophages have received considerable attention (Denardo and Ruffell, 2019). In solid tumors, such as gastric cancer and melanoma, high levels of M2 macrophage infiltration are associated with higher expression levels of immune checkpoints, such as programmed cell death protein 1 (PD-1) and programmed death-ligand 1 (PD-L1), suggesting that macrophages can be used as a potential therapeutic target for tumors (Ju et al., 2020; Kim et al., 2020). In tumor therapy, the use of anti-M2 macrophages combined with immune checkpoint inhibitors improves the therapeutic effect and provides a new idea for the treatment of tumors (Gordon et al., 2017).

With the development of high-throughput sequencing technology, patients with cancer are classified into different subtypes according to the expression of specific genes; thus, individualized treatment regimens based on the characteristics of different subtypes provide a new direction to improve the prognosis of patients (Cook and Vanderhyden, 2019). Based on the aforementioned information, we believe that immune infiltration is a good starting point. From the perspective of immune cell infiltration, this study applied the Cibersort algorithm to analyze the infiltration of M2 macrophages in patients with colon cancer, and divided colon cancer into two subtypes by screening Hub genes related to M2 macrophages. Finally, M2 macrophage infiltration score (M2I Score) was determined to accurately predict the prognosis of patients and their sensitivity to immunotherapy, and to provide some reference for the clinical individualized medication diagnosis and treatment.

## Materials and Methods

### Data Collection

The transcriptome data (RNA-seq; Fragments Per Kilobase Million value) and related clinical information of patients with colon adenocarcinoma (TCGA-COAD) were downloaded from The Cancer Genome Atlas (TCGA) database.^1^ The R-package “LIMMA” was used to normalize transcriptome data. Transcriptome data from multiple samples from the same patient were deleted. The chip data of colon cancer patient numbered GSE39582 in the Gene Expression Omnibus (GEO) database^2^ was selected as the verification set. The validation set was selected according to the following criteria: (a) with mRNA expression data, and (b) with complete patient prognosis information. Data from the GEO database were used to verify the conclusions of the TCGA database analysis.

### Analysis of M2 Macrophage Infiltration and Identification of Related Genes

The Cibersort algorithm was used to analyze the level of infiltration of 22 kinds of immune cells in TCGA patients with colon cancer. CIBERSORT,^3^ which is based on the principle of the linear support vector regression on immune cell subtype of deconvolution of the expression of matrix, is a tool that can be used to estimate the immune cell infiltration. Combined with the overall survival time of patients, the effect of M2 macrophage infiltration level on survival was analyzed using the R pack “Survival.”

The weighted gene co-expression network analysis (WGCNA) algorithm was used to define the genes associated with M2 macrophages in colon cancer. WGCNA is a method to construct gene co-expression network based on gene expression data. First, the first 5,000 genes after the mean absolute deviation sequencing were selected, and the R package of WGCNA was used to construct the co-expression network of mRNA expression of the above genes. A soft threshold parameter β was then chosen to construct a proximity matrix that matched the gene distribution to a connection-based scale-free network. Then, the adjacency was transformed into a topological overlap matrix (TOM), and the linkage hierarchical clustering was performed according to the average of different measures based on TOM. In the end, a gene-tree with a minimum (genome) of 30 and a module tree with a cut line of 0.25 was chosen, combined with several modules to produce more rigorous results.

After the WGCNA analysis, a correlation analysis was conducted between the M2I Score and the co-expression modules enriched by WGCNA. According to the gene expression in the module and the data of immune cell infiltration of patients, “cor” function in R package WGCNA was used to calculate the correlation between module and trait. Depending on the correlation, we obtained a module most associated with M2 macrophages. Subsequently, the genes in the co-expression module were enriched and analyzed through the clusterProfiler package to explore the biological functions of the genes in the module.

### Screening of Hub Genes

Consensus Clustering is an algorithm that can be used to identify the members and number of clusters in a dataset. In this study, Consensus ClusterPlus package was used to conduct the conformance cluster analysis on the selected genes in the co-expression modules, and Kaplan–Meier survival analysis was used to judge the difference of survival time among different clusters. CytoHubba was used to analyze the co-expression network of genes in the module, and the top 20 nodes were screened for further analysis. Univariate Cox regression analysis was used to screen the prognostic genes in above 20 genes, followed by 1,000 times of Lasso analysis to select the most stable genes as hub genes. Finally, conformance cluster analysis was performed on the finally screened hub genes again to determine the survival differences between different clusters.

### Validation of Selected Hub Genes

First, the reliability of the clustering analysis method was verified. Principal component analysis (PCA) was used to verify the results of the previous concordant analysis, to prove that the hub gene that was identified could efficiently divide patients with colon cancer into two categories. Subsequently, single-sample gene set enrichment analysis (ssGSEA) was used to calculate the scores of immune cell infiltration in TCGA-COAD patients, and the differences of macrophage scores among different clusters were analyzed. The data of ssGSEA signature genes was downloaded from GSEA database^4^. Then, in order to verify the reliability of the data set, the GSE39582 data set was selected from the GEO database to verify the grouping based on hub genes and the prognostic correlation. So far, the stability of the results has been verified in different clustering algorithms and different datasets.

### Generation of M2I Score and the Difference Between High and Low Rating Groups

According to the previous typing results, a gene was classified as gene signature A when the gene expression decreased with the increase of typing value, whereas it was classified as gene signature B if the gene expression increased with the increase of typing value. PCA algorithm was used to calculate the M2I Score of each sample. The calculation formula is as follows:

M2I⁢Score=Σ⁢PC1A-Σ⁢PC1B

The optimal truncation value was calculated according to the survival status of the patients, and the patients were divided into the high-rating group and the low-rating group, and the differences in survival status between the high-rating group and the low-rating group were analyzed. Meanwhile, the differences in immune checkpoints and M2 macrophage marker gene expression between the different rating groups were analyzed by Wilcoxon test.

### Relationship Between M2I Score and Somatic Variation

The mutation data of TCGA-COAD patients were downloaded and Perl was used to count the number of non-synonymous mutations. The total number of somatic gene coding errors, base substitutions, gene insertion or deletion errors detected per million bases were defined as the tumor mutation load (TMB). The difference of the TMB between the high- and low-rating groups was calculated, and the *P*-value (*P* < 0.05) was statistically significant. Spearman correlation coefficient was used to analyze the relationship between the M2I Score and TMB. Next, the R package “maftools” was used to identify the COAD driver genes, and the status of the top 20 genes with the highest mutation frequency in the high rating group and the low rating group was further analyzed.

### Differences in MSI and Immunotherapy Sensitivity Between the High and Low Rating Groups

Data on MSI and immunotherapy sensitivity of TCGA-COAD patients were downloaded from The Cancer Immunome Atlas database^5^. The Cancer Immunome Atlas database (TCIA) was developed and maintained by the Institute of Bioinformatics. The database can query data on gene expression of specific immune-related gene sets, cell composition of immune infiltrates, and tumor heterogeneity. The difference in the M2I Score among patients with different levels of MSI (MSS, MSI-L, and MSI-H) and the proportion of different levels of MSI between groups with high and low M2I Scores were analyzed. Subsequently, according to the sensitivity scores of TCGA-COAD patients to PD-L1 and CTLA4 inhibitors in the TCIA database, the differences in sensitivity to immunotherapy between the groups with high- and low-ratings were also analyzed.

### Comparison of Predictive Abilities Between M2I Score and Cibersort M2 Macrophage Score

Consistent with previous methods, we analyzed the relationship between Cibersort M2 macrophage score and TMB, MSI levels and immune checkpoint inhibitor sensitivity in colon cancer patients. Then we compared the results with the results of M2I score.

## Results

### Data Downloading and Collection

A total of 432 patients with colon cancer from the TCGA dataset were included in this study. The validation set was from the GEO database. The data set GSE39582 included 566 samples of colon cancer tissues and 19 samples of paracancerous normal tissue. The cancer tissue samples were selected for further analysis. TCGA and GEO related patient information is shown in Supplementary Table 1.

### Immunoinfiltration Analysis and Screening of M2 Macrophage Related Genes in COAD Using WGCNA

**FIGURE 1:**
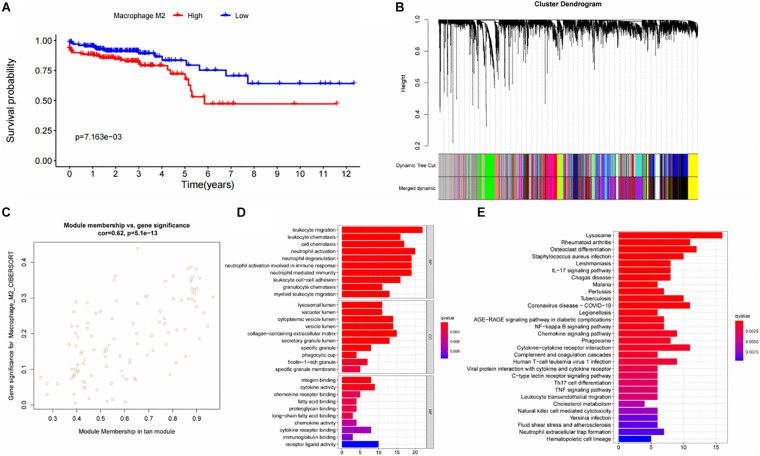
Analysis and screening of M2 macrophage related genes. **(A)** Survival analysis of patients with different levels of M2 macrophage infiltration. **(B)** Analysis of gene distribution in WGCNA network. **(C)** Correlation analysis of Tan module and M2 macrophages. **(D)** GO analysis of genes in the Tan module. **(E)** KEGG analysis of genes in the TAN module.

We used Cibersort to analyze the immune cell infiltration in TCGA-COAD patients, and obtained the M2I Score of each sample. Survival analysis showed that the infiltration level of M2 macrophages had an impact on the survival time of patients (Figure 1A). Patients with higher M2I Scores had poorer survival (*P* = 7.163e-03). To further identify the genes associated with M2 macrophage infiltration in colon cancer, we performed WGCNA analysis on the data and found that when the soft threshold was set to 7, it accorded with the scale-free property of biological network; hence, we used β = 7 to construct the weighted network. Then, we carried out the average linkage hierarchical clustering, identified the modules based on TOM’s differences, dynamic tree pruning and merging processing, and obtained a total of 20 meaningful modules with different colors (Figure 1B). Then, we correlated all the modules analyzed in WGCNA with the M2I Scores, and found that the tan module had the strongest correlation with M2 macrophages (*R* = 0.62) and *P* = 6.1e-13 was statistically significant (Figure 1C). We enriched and analyzed 110 genes in the tan module, and found that the biological functions of this group of genes were related to immunity (Figures 1D,E). So far, we found a group of stable genes related to M2 macrophages in intestinal cancer, whose biological functions were correlated with tumor immunity to a certain extent.

### Screening of Hub Genes Based on Cluster Analysis

**FIGURE 2:**
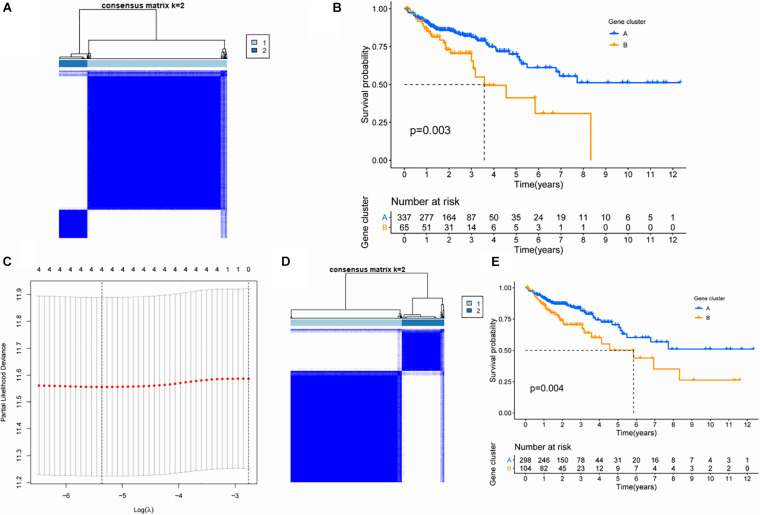
Screening of key genes based on cluster analysis. **(A)** Consistency cluster analysis based on genes within the TAN module. **(B)** Survival analysis of patients between subgroups differentiated by genes within the TAN module. **(C)** Lasso analysis and screening of hub gene. **(D)** Consistency cluster analysis based on four hub genes. **(E)** Survival analysis of patients between subgroups differentiated by hub gene.

In order to further explore the characteristics of genes in the TAN module, we performed K-value based consistent clustering based on the expression of 110 genes involved in the tan module. According to the cumulative distribution function (CDF), *k* = 2 was selected as the optimal parameter, and TCGA-COAD patients were divided into two groups, namely, ClusterA and ClusterB (Figure 2A). Further, through survival analysis, it was found that the overall survival (OS) of patients in ClusterA and ClusterB were significantly different (*P* = 0.003), as shown in Figure 2B. Thus, we inferred that M2 macrophage-related genes in the TAN module can affect the OS of patients with colon cancer through immune-related pathways.

In order to screen hub genes in the module, CytoHubba was used to analyze the co-expression network of genes in the TAN module, and the top 20 nodes were screened for further analysis (Supplementary Table 2). The network diagram of the above 20 nodes was shown in Supplementary Figure 1. Univariate Cox analysis was conducted on the genes in the above 20 genes to screen the genes related to the survival of patients. When the *P*-value in univariate Cox analysis was < 0.05, five candidate genes could be screened (Table 1). Furthermore, through 1000 LASSO analyses, four hub genes (Figure 2C) were finally identified, namely: ankyrin repeat and sterile alpha motif domain containing 4B (ANKS4B), Cathepsin D (CTSD), tissue inhibitor of metalloproteinase 1 (TIMP1), and zinc finger protein 703 (ZNF703), which are the most stable prognostic-related genes associated with M2 macrophages in colon cancer.

**TABLE 1:** Results of the univariate Cox regression analysis between gene expression and OS.

ID	HR	HR.95L	HR.95H	*P*-value
ANKS4B	0.745	0.575	0.965	0.026
TIMP1	1.421	1.102	1.831	0.007
CTSD	1.479	1.062	2.061	0.021
NFKB2	1.437	1.022	2.019	0.037
ZNF703	0.773	0.604	0.988	0.040

To verify the prognostic value of the four hub genes, according to the expression of these four genes, we applied the consistency cluster analysis based on the *K* value, and selected *k* = 2 as the optimal parameter. TCGA-COAD patients were divided into two groups, named ClusterA and ClusterB (Figure 2D). Survival analysis showed that the OS of ClusterB patients was significantly lower than that of ClusterA patients (*P* = 0.004) (Figure 2E).

### Validation of Screened Hub Genes

**FIGURE 3:**
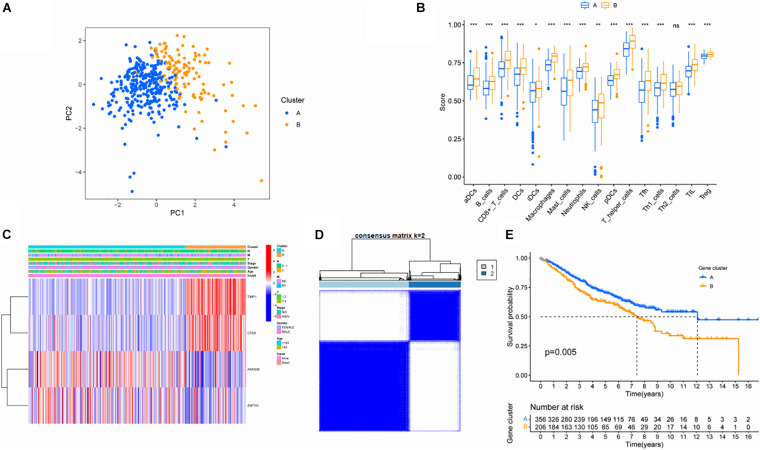
Validation of screened key genes. **(A)** PCA diagram of the TCGA queue. **(B)** Comparison of ssGSEA scores among different subgroups. **(C)** Distribution of hub gene among different subgroups. **(D)** Consistent clustering analysis based on four hub genes in GSE39582. **(E)** Survival analysis of patients in different subgroups in the GEO cohort.

In order to verify the stability of the consistency clustering method based on K value for the classification of TCGA-COAD patients, another classification method, namely PCA analysis method, was selected for verification again. The results are shown in Figure 3A. Meanwhile, ssGSEA results showed that the macrophage score of ClusterB was significantly higher than that of ClusterA (*P* < 0.001; Figure 3B). The expression of the four hub genes in different clusters is shown in Figure 3C. At the same time, another dataset, GSE39582, was also chosen to prove that the change of the dataset would not affect the conclusion. In GSE39582, cluster analysis was conducted according to the expression of hub genes, and the results showed that these four genes could efficiently divide patients with colon cancer into two clusters (Figure 3D). The survival analysis between the two groups showed statistically significant differences in OS (Figure 3E). Since then, consistent results in different datasets and different classification methods were obtained, proving the stability and accuracy of the above key gene screening.

### Determination of the M2I Score

**FIGURE 4:**
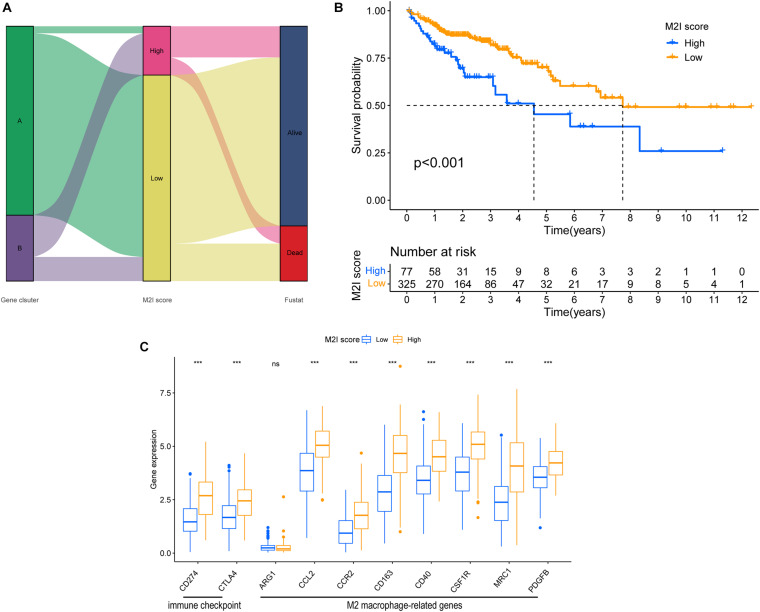
Construction of the M2I score. **(A)** Sankey plot of survival outcomes in the distribution set of M2I scores in different subgroups. **(B)** Survival difference between groups with high and low M2I rating. **(C)** Immune checkpoint related genes and M2 macrophage related genes were expressed in the subgroups with high and low M2I scores.

In order to determine the M2I Score in TCGA-COAD patients, the PC1 values of genes in gene signature A and B were calculated by PCA, and the sum of PC1 (SPC1a and SPC1b) of gene signature A and B were calculated. Subsequently, the difference between SPC1A and SPC1B was used as M2 macrophage score (M2I Score). Patients in the TCGA cohort were divided into two groups according to the M2I Score by using the optimal cut-off value obtained by X-tile software. Figure 4A shows the distribution of patients with high and low scores. Patients with high scores were mainly from ClusterB, whereas those with low scores were mainly from ClusterA. Simultaneously, survival analysis showed that the survival of patients in the high-rated group was significantly worse than that in the low-rated group (*P* < 0.001) (Figure 4B). Meanwhile, according to the Wilcoxon test, immune checkpoints (CD274 and CTLA4) and M2 macrophage marker genes (CCl2, CCR2, CD163, CD40, CSF1R, MRC1, and PDGFB) were significantly overexpressed in the high M2I Score group (Figure 4C). At this point, there was an accurate M2I Score, which reflects the level of immune checkpoints and M2 macrophage marker genes.

### Correlation Between the M2I Score and Somatic Variation

**FIGURE 5:**
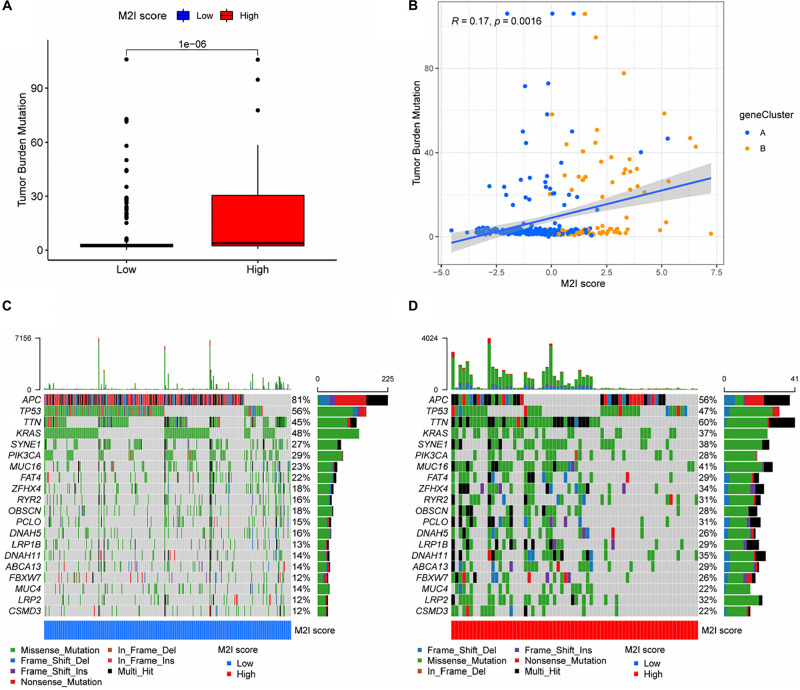
Correlation between M2I Score and somatic variation. **(A)** TMB difference between high and low M2I score subgroups. **(B)** Correlation analysis between M2I score and mutation load. **(C,D)** oncoPrint of the top 20 genes with the highest mutation frequency in the subgroup of high and low M2I scores.

There is substantial evidence that a higher TMB represents a better patient response to immunotherapies, such as immune checkpoint inhibitor therapy. Considering the important clinical significance of TMB, we decided to explore the relationship between M2I Score and TMB. For this reason, we first analyzed the differences in the TMB values between groups with high and low M2I Scores, and the results showed that TMB was significantly higher in the group with high scores than in the group with low scores (*P* = 1e-06; Figure 5A). Meanwhile, Spearman correlation analysis showed that the M2I Score was positively correlated with TMB (*R* = 0.17, *P* = 0.0016; Figure 5B). In addition, we also analyzed the differences of somatic cell variation driver genes in the high and low M2I Score group of colon cancer. The top 20 driver genes with the highest frequency of change were selected using the R package “Maftools” for analysis, and the mutation frequency of 16 genes in the high-rated group was higher than that in the low-rated group (Figures 5C,D). These results suggest that patients in the high-rated group may have a better response to immunotherapy.

### Role of the M2I Score in Predicting the Benefit of Immunotherapy

**FIGURE 6:**
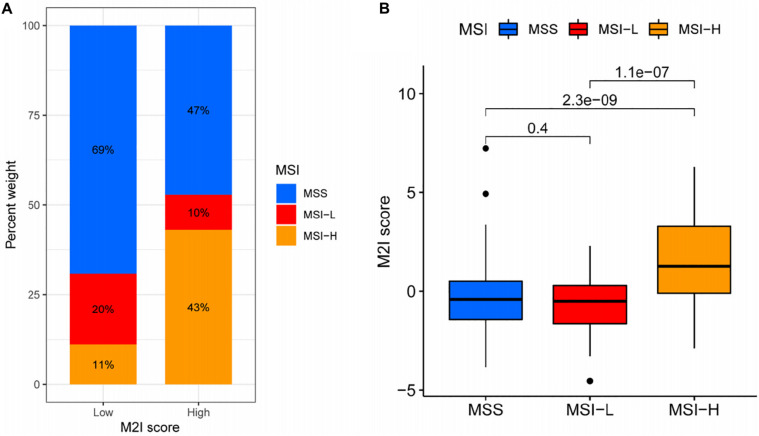
Relationship between M2I score and MSI. **(A)** The proportion of different MSI levels in the subgroups with high and low M2I scores. **(B)** Differences in M2I scores among groups with different MSI levels.

In colon cancer, higher MSI often represents patients’ ability to obtain better immunotherapeutic effects (Ganesh et al., 2019). In order to further explore the relationship between M2I Score and MSI, relevant data from the TCIA database were used for analysis. According to the TCIA database, the MSI of patients with TCGA-COAD is divided into three levels: (1) MSS – microsatellite stabile; (2) MSI-L – low instability of microsatellites; and (3) MSI-H – high instability of microsatellites. The proportion of patients with the three kinds of MSI levels in the high and low M2I Score group was calculated. The results showed that the proportion of MSI-H in the high M2I Score group was (43%) higher than that in the low M2I Score group (11%), and the chi-square test showed that the difference was statistically significant (Figure 6A). Meanwhile, patients were grouped according to the MSI level, and the differences of M2I Score among different groups were compared. The results showed that the M2I Score of patients in the MSI-H group was higher than that of patients in the MSI-L and MSS groups (*P* = 1.1e-07 and 2.3e-09, respectively) (Figure 6B). This suggests that patients with high M2I Scores are more likely to benefit from immunotherapy.

**FIGURE 7:**
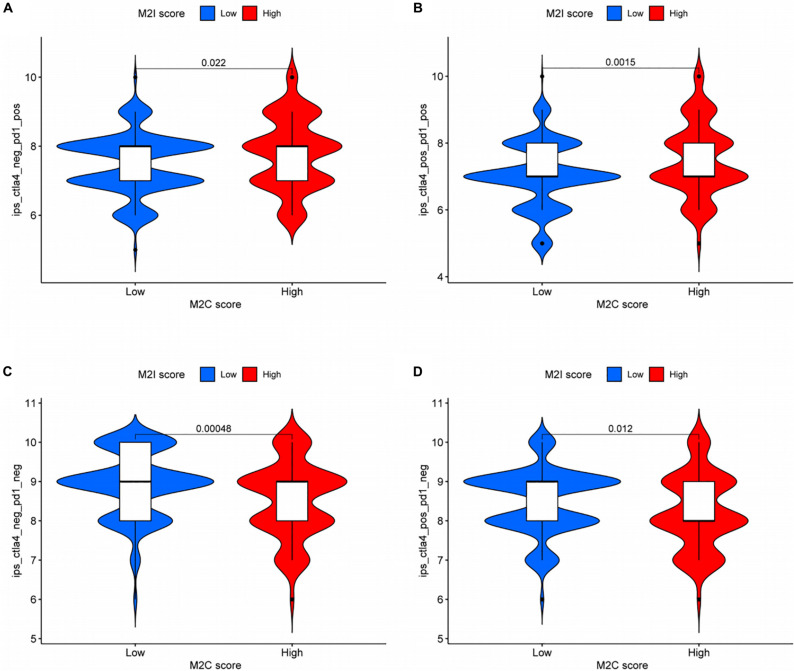
Role of M2I score in predicting immunotherapy benefits. **(A–D)** Sensitivity of patients with high and low M2I score subgroups to four treatments [**(A)** use of PD-1 inhibitor alone; **(B)** use of PD-1 inhibitor in combination with CTLA-4 inhibitor; **(C)** do not use immune checkpoint inhibitors; and **(D)** CTLA-4 inhibitor alone].

Based on the above results, we analyzed the difference of efficacy between PD-1 inhibitor and CTLA4 inhibitor in patients with different rating groups according to the sensitivity data of immunotherapy in the TCIA database. The results showed that patients in the M2I high-level group, previously associated with somatic variation and MSI analysis, who could be more sensitive to immunotherapy, were more sensitive to PD-1 inhibitors (*P* = 0.022, Figure 7A) and PD-1 inhibitors in combination with CTLA4 inhibitors (*P* = 0.0015, Figure 7B). For the group with low sensitivity to immunotherapy, we did not use immune checkpoint inhibitors (*P* = 0.00048, Figure 7C) or CTLA4 inhibitors alone (*P* = 0.012, Figure 7D), which may achieve better efficacy. Taken together, these data suggest that M2I Score may be associated with immunotherapeutic response and may have implications for the selection of immunosuppressive agents in clinical treatment.

### Comparison of Predictive Ability Between M2I Score and Cibersort M2 Macrophage Score

Survival analysis showed that both Cibersort M2 macrophage score (*P* = 7.163E-03) and M2I score (*P* < 0.001) were associated with patients’ OS (Figures 1A, 4B), and M2I had a smaller *P*-value. In addition, there was no correlation between Cibersort M2 macrophage score and TMB of patients (*R* = 0.013, *P* = 0.8), and there was no difference in TMB between high and low Cibersort M2 macrophage score groups (*P* = 0.76) (Supplementary Figures 2A,B). MSI correlation analysis showed that there was no statistically significant difference in Cibersort M2 macrophage score among patients in MSI-H, MSI-L and MSS groups (Supplementary Figure 2D). Meanwhile, Chi-square test showed that, there was no difference in the proportion of MSI-H patients between high and low Cibersort M2 macrophage score groups (*P* = 0.1927) (Supplementary Figure 2C). The differences in sensitivity of patients to immune checkpoint inhibitors between high and low Cibersort M2 macrophage score groups were compared, and the results showed that Cibersort M2 macrophage score could not reflect the sensitivity of patients to immune checkpoint inhibitors (Supplementary Figures 3A–D). Therefore, we believe that compared with Cibersort M2 macrophage score, M2I score has better predictive ability.

## Discussion

As one of the most common malignancies, colon cancer has been the third most common cancer among new cases and the second most common cause of cancer-related deaths in 2020. Therefore, developing more effective treatments for colon cancer has been an urgent problem for researchers (Siegel et al., 2020). As the most abundant immune cells in colon, macrophages play an important role in the interaction between tumor cells and tumor microenvironment. As one of the carcinogenic differentiation types, M2 macrophages have attracted extensive attention as a potential therapeutic target (Badawi et al., 2015). In this study, we developed a method to quantify M2 macrophage infiltration in CRC. Our results indicate that M2 macrophage infiltration score can accurately predict patient survival and has potential guiding significance for the selection of immunotherapy drugs.

An increasing number of studies have shown that M2 macrophages are involved in the occurrence and development of colon cancer, promoting its invasion and metastasis, and thus leading to poor prognosis of patients. Therefore, it is very important to determine a score that can evaluate the infiltration degree of M2 macrophages in patients with colon cancer (Vinnakota et al., 2017; Lan et al., 2018). In this study, immune cell infiltration analysis was performed on TCGA-COAD samples according to Cibersort algorithm, and WGCNA was used to identify modules associated with M2 macrophage infiltration. Consistency clustering based on the genes within the module could divide the patients into two clusters with different survival conditions. Subsequently, univariate and Lasso Cox regression analyses were performed to screen the most robust prognostic biomarkers to establish the M2-macrophage-related genetic signature. Finally, four hub genes were obtained: ANKS4B, CTSD, TIMP1, and ZNF703. ANKS4B is expressed in intestinal cells and is distinct in the distal part of the brush-like microvilli. Studies have shown that this gene plays an important role in the assembly of brush-like microvilli (Graves et al., 2020). Destruction or malformation of the brush border is associated with a number of intestinal diseases, including infections of attached and disappearing microorganisms and microvillous inclusion diseases (Vallance et al., 2002; Wilson et al., 2016). In addition, the loss of brush-limbic proteins involved in cell polarity in colon cancer is important for tumor development (Rocco et al., 2012), suggesting that ANKS4B may influence the occurrence and development of colon cancer. Proteins encoded by CTSD are involved in a variety of immune-related biological processes, such as antigen processing and exogenous antigen presentation (Benes et al., 2008). HPA database showed that CTSD expression was significantly up-regulated in tumor tissues. In tumor research, CTSD secreted by tumor cells into the extracellular space plays an important role in the invasion and metastasis of breast cancer and ovarian cancer (Pranjol and Whatmore, 2020). In colon cancer, activation of the Wnt/β-catenin signaling pathway can lead to increased levels of endogenous CTSD, and thus enhance the proliferation and invasiveness of colon cancer cells (Basu et al., 2019). The proteins encoded by TIMP1 may inhibit the activity of matrix metalloproteinases and regulate the balance of matrix remodeling during extracellular matrix degradation (Batra et al., 2012). In colon cancer, TIMP1 has higher expression levels in tumor tissues and lymph node metastases than in normal tissues, the expression level of TIMP1 in colon cancer is significantly positively correlated with CD4^+^T cells, macrophages, neutrophils and dendritic cells. Studies have shown that TIMP1 affects the prognosis of patients through the FAK-PI3K/AKT and MAPK pathways (Song et al., 2016). ZNF703 plays an important role in the occurrence and development of head and neck squamous cell carcinoma, non-small cell lung cancer and other tumors (Baykara et al., 2016; Orhan et al., 2019). In colon cancer, ZNF703 inhibition can hinder the proliferation and migration of colorectal cancer cells, which is considered as a potential therapeutic target for metastatic colon cancer (Ma et al., 2014).

Based on these four hub genes, patients with colorectal cancer were divided into two groups, Cluster A and Cluster B, by the consistent clustering method, and the results showed that the prognosis of the two groups was significantly different. At the same time, ssGSEA was used to analyze the immune cell infiltration in patients, and the results showed that there were also significant differences in the infiltration of macrophages between the two groups. Thus, we concluded that these four hub genes were related to M2 macrophages in CRC patients, and could affect the OS of patients. In addition, the external verification set from the GEO database also verified the accuracy of the genetic signature constituted by the four hub genes in predicting the prognosis of patients.

Considering the individual differences in immune cell infiltration, it is important to quantify the infiltration pattern of M2 macrophages in individual tumors. Individual-based models based on tumor subtype specific biomarkers have been well used in breast cancer and other cancers to improve the accuracy of patient prognosis prediction (Callari et al., 2016). In this study, the above hub gene was use as a potential “subtype biomarker,” and established an M2I score to quantify M2 macrophage infiltration in each sample. Survival analysis showed that higher M2I scores were associated with poorer survival. Ju et al. have shown that tumor-associated macrophages can induce tumor cells to express PD-L1 through IL-6 and TNF-α signaling (Ju et al., 2020). In colon cancer, macrophages are present in higher concentrations in patients with DMR-MSI-H, which represents good immunotherapeutic sensitivity (Hu et al., 2019; Narayanan et al., 2019). Based on the above conclusions, we decided to further explore the association between M2I score and immunotherapy sensitivity. Numerous studies have shown a clear association between gene mutations and the patients’ responsiveness to immunotherapy, such as immune checkpoint inhibitor therapy (Pan et al., 2020). In this study, we found that the TMB was significantly increased in patients with higher M2I Scores, and the mutation frequency of several genes was also different between the high and low M2I rating groups. While the correlation between the M2I Score and TMB was not very strong (0.17), this may be due to the insufficient sample size and the fact that the data came from a single database. Further analysis of the relationship between the M2I Score and MSI also showed that higher M2I Score also represented higher MSI level. Therefore, we preliminarily concluded that M2I Score may be associated with immunotherapy sensitivity. Finally, according to the relevant data in the TCIA database, the sensitivity differences of patients with low and low M2I ratings to PD-1 and cytotoxic T-lymphocyte–associated antigen 4 (CTLA-4) inhibitors were analyzed. We found that patients with high M2I Score had higher sensitivity to PD-1 inhibitors and PD-1 inhibitors combined with CTLA4 inhibitors. For patients in the low-rated group, no immune checkpoint inhibitors or CTLA4 inhibitors alone may yield better results. According to Fiegle et al. (2019), dual inhibition of CTLA-4 and PD-L1 resulted in tumor growth arrest and complete blocking of liver metastasis, whereas inhibition of CTLA-4 and PD-L1 alone only modestly reduced metastatic spread of colon cancer cells. In this context, we conclude that dual immune checkpoint suppressive therapy for CTLA-4 and PD-L1 may be the preferred immunotherapy for patients with high M2I scores.

In order to prove the superiority of M2I Score, we compared the predictive results of M2I Score and Cibersort M2 macrophage Score on OS, MSI, TMB and immunotherapy sensitivity of patients. The results show that M2I Score has better predictive ability in the above four aspects, which also proves the rationality and accuracy of our selection of M2I Score instead of Cibersort M2 macrophage Score in subsequent studies.

In general, four hub genes associated with M2 macrophages in colon cancer were screened out in this study, which could affect patients’ OS through immune-related pathways. Based on these four genes, we determined an M2I Score which could predict patients’ survival. In addition, we hypothesized that these four genes may be involved in the sensitivity of colon cancer patients to immune checkpoint inhibitors. There are still some limitations in this paper, which are mainly reflected in the fact that this study is based on the exploration of public database, and thus, experimental verification is still needed. Furthermore, the specific regulatory mechanism of characteristic genes on colon cancer still needs to be explored.

## Conclusion

M2 macrophage infiltration is associated with poor prognosis in colon cancer. Four prognostic hub genes associated with M2 macrophages in colon cancer were identified as follows: ANK4B, CTSD, TIMP1, and ZNF703, and the stability of these results was verified by different clustering methods and GSE39582 dataset. The M2I Score was determined and the patients with colon cancer were divided into two subgroups: high M2 Score group and low M2 Score group. The correlation between the M2I Score and somatic cell variation and MSI was analyzed. The results showed that in the high-rated group, the TMB was higher, MSI was stronger, and immunotherapy was more sensitive. Combined with the above results and the TCIA database, we conclude that patients in the high-rated group, who are more sensitive to immunotherapy, should be prioritized for therapy with PD-1 inhibitors or PD-1 inhibitor combined with CTLA-4 inhibitors, whereas patients in the low-rated group should be prioritized for therapy with no immune checkpoint inhibitors or with CTLA4 inhibitors alone.

## Data Availability Statement

The original contributions presented in the study are included in the article/Supplementary Material, further inquiries can be directed to the corresponding authors.

## Author Contributions

YA and MS conceived the study. BX conducted all the statistical analyses. ZP and YA reviewed relevant literature and drafted the manuscript. NW, GY, MC, and XY provided guidance to the study. All authors read and approved the final manuscript.

## Conflict of Interest

The authors declare that the research was conducted in the absence of any commercial or financial relationships that could be construed as a potential conflict of interest.

## Publisher’s Note

All claims expressed in this article are solely those of the authors and do not necessarily represent those of their affiliated organizations, or those of the publisher, the editors and the reviewers. Any product that may be evaluated in this article, or claim that may be made by its manufacturer, is not guaranteed or endorsed by the publisher.

## Footnotes

1
https://portal.gdc.cancer.gov/

2
https://www.ncbi.nlm.nih.gov/geo/

3
https://cibersort.stanford.edu/

4
http://www.gsea-msigdb.org/

5
https://tcia.at/

## Abbreviations

COAD: colon adenocarcinoma
CTLA-4: cytotoxic T-lymphocyte –associated antigen 4
GEO: Gene Expression Omnibus
M2I: M2 macrophage infiltration
MSI-H: group with high instability of microsatellites
MSI-L: group with low instability of microsatellites
MSS: microsatellite stability
PD-1: programmed cell death protein 1
PD-L1: programmed death-ligand 1
ssGSEA: single-sample gene set enrichment analysis
SPC: sum of PCI
TCGA: The Cancer Genome Atlas
TCIA: The Cancer Immunome Atlas
Th: T helper cell
TMB: tumor mutation load
TOM: topological overlap matrix
WGCNA: weighted gene co-expression network analysis.
