# Supplementary figures and images for: Establishment and Validation of a Genetic Label Associated With M2 Macrophage Infiltration to Predict Survival in Patients With Colon Cancer and to Assist in Immunotherapy
Source: Front Genet. 2021 Sep 6;12:726387. doi: 10.3389/fgene.2021.726387 (PMC8451970; doi:10.3389/fgene.2021.726387)

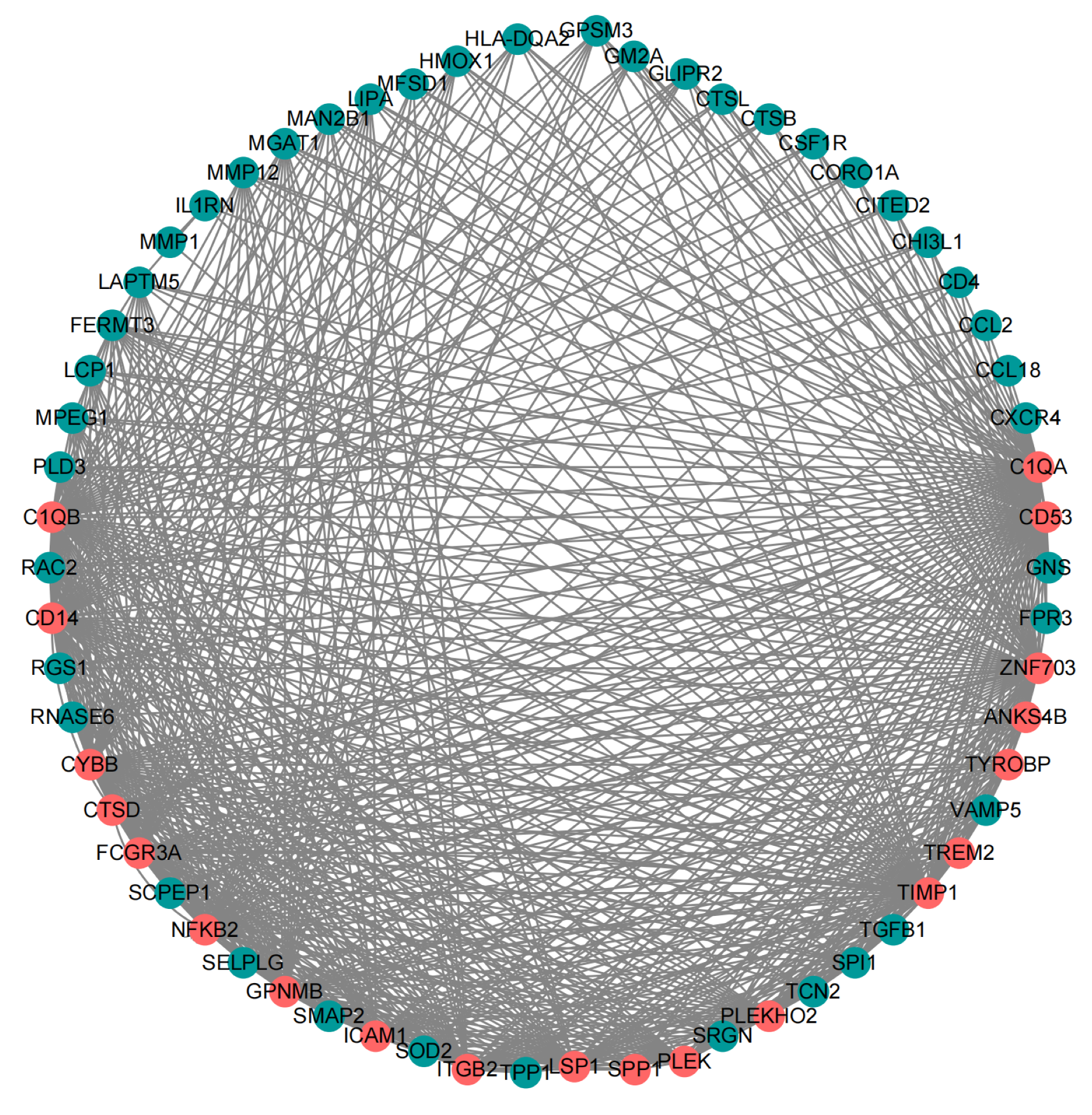

Supplement: Supplementary file 1 [file Image_1.TIF]

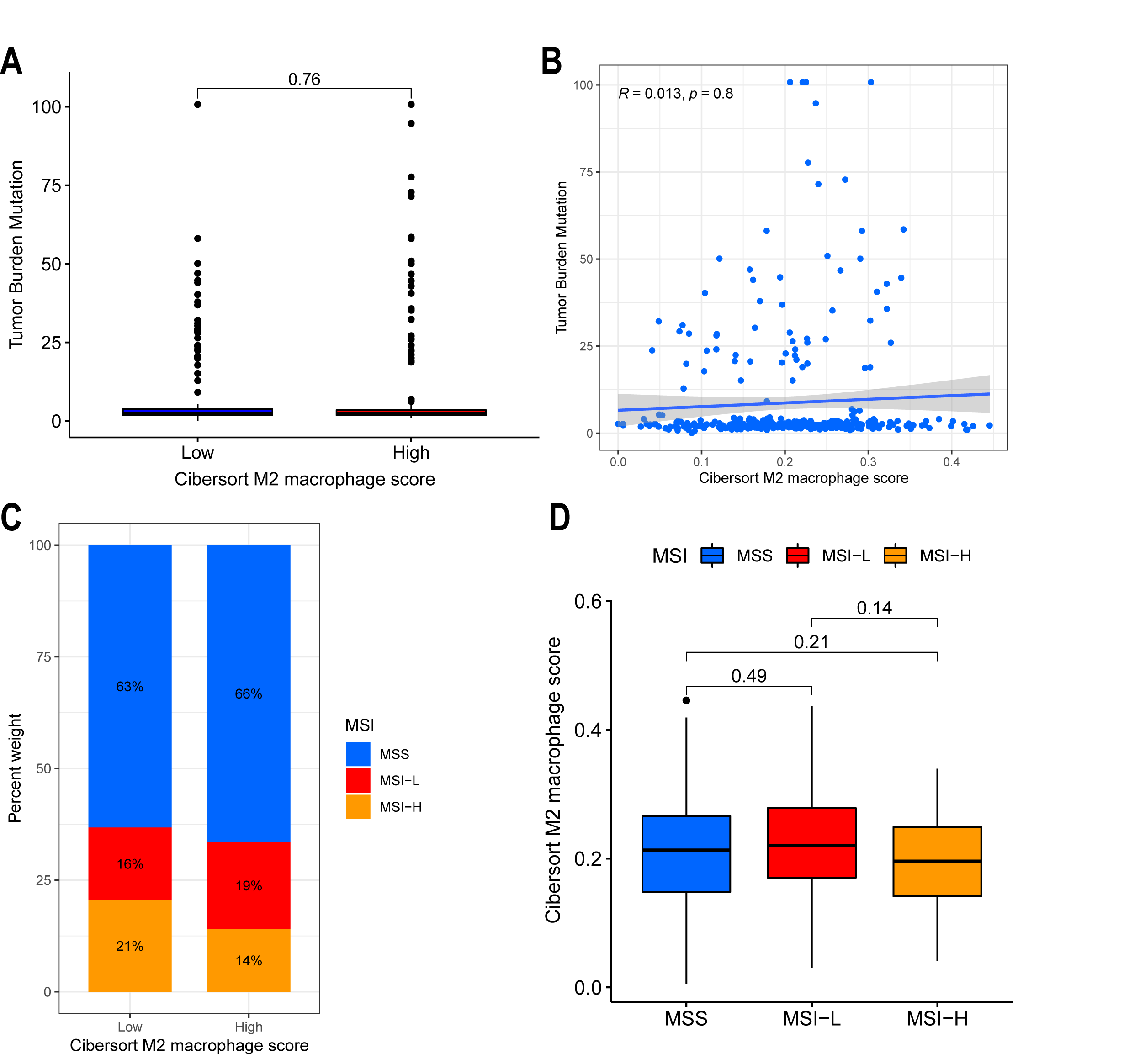

Supplement: Supplementary file 2 [file Image_2.TIF]

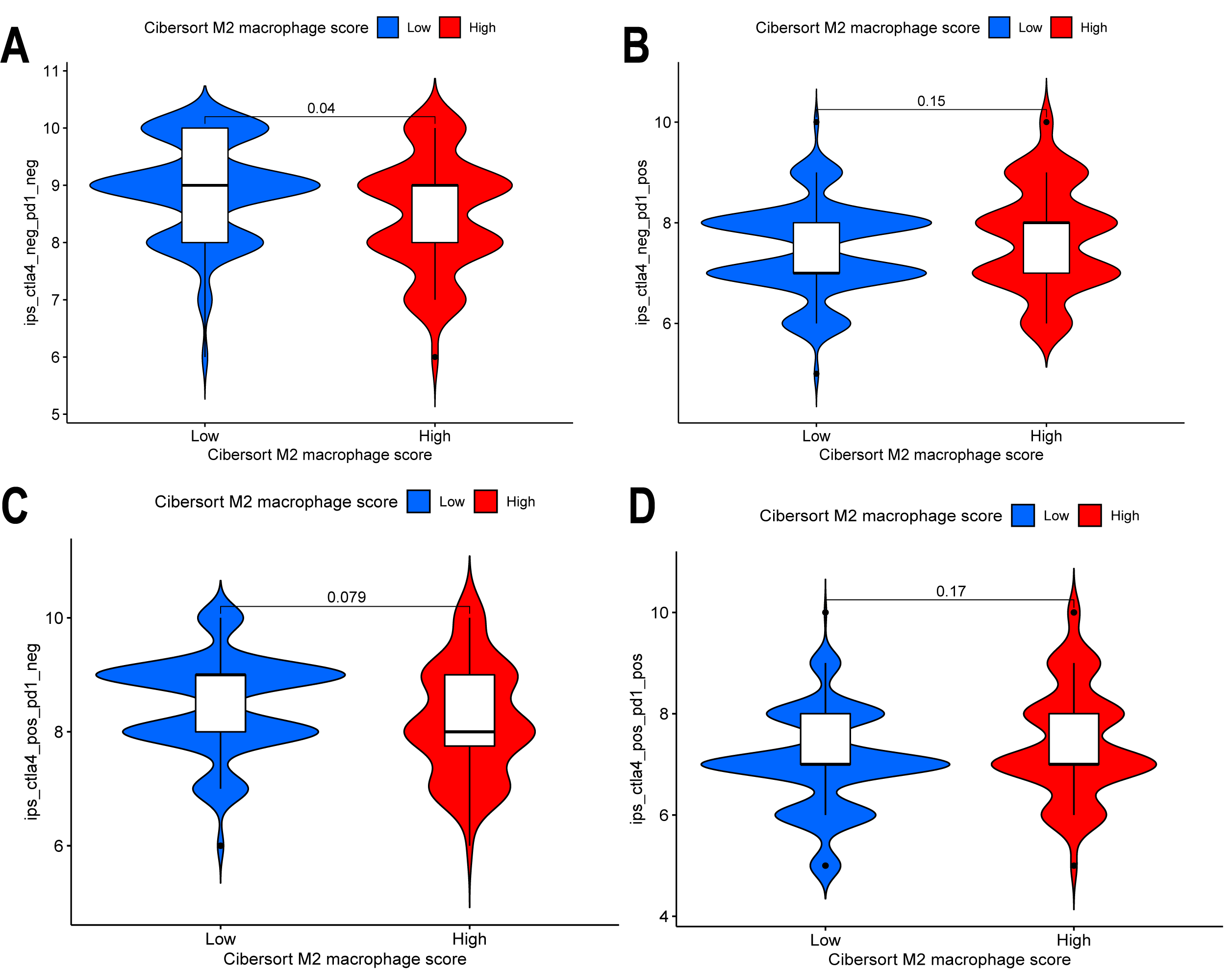

Supplement: Supplementary file 3 [file Image_3.TIF]
