# Supplementary material for: Establishment and Validation of a Genetic Label Associated With M2 Macrophage Infiltration to Predict Survival in Patients With Colon Cancer and to Assist in Immunotherapy
Source: Front Genet. 2021 Sep 6;12:726387. doi: 10.3389/fgene.2021.726387 (PMC8451970; doi:10.3389/fgene.2021.726387)
Supplement: Supplementary file 4 [file Table_1.DOCX]

Supplementary Material

**Table S1 Clinical characteristics of the colon cancer patients used in this study**

|  | **TCGA-COAD** | **GSE39582** |
| --- | --- | --- |
| **No. of patients** | 432 | 566 |
| **Age (median, range)** | 68 (31-90) | 68.5 (22-97) |
| **Gender (%)** |  |  |
| Female | 203 (47.0%) | 256 (45.2%) |
| Male | 229 (53.0%) | 310 (54.8%) |
| **Stage (%)** |  |  |
| 0 | NA | 4 (0.7%) |
| I | 72 (16.7%) | 33 (5.8%) |
| II | 169 (39.1%) | 264 (46.6%) |
| III | 120 (27.8%) | 205 (36.2%) |
| IV | 60 (13.9%) | 60 (10.6%) |
| unknown | 11 (2.5%) | NA |
| **T (%)** |  |  |
| T1 | 10 (2.3%) | 11 (1.9%) |
| T2 | 73 (31.5%) | 45 (8.0%) |
| T3 | 294 (68.1%) | 367 (64.8%) |
| T4 | 54 (12.5%) | 119 (21.0%) |
| Tis | 1 (0.2%) | 4 (0.7%) |
| unknown | NA | 20 (3.5%) |
| **N (%)** |  |  |
| N0 | 256 (59.2%) | 302 (53.4%) |
| N1 | 100 (23.1%) | 134 (23.7%) |
| N2 | 76 (17.6%) | 98 (17.3%) |
| N3 | NA | 6 (1.1%) |
| unknown | NA | 26 (4.6%) |
| **M (%)** |  |  |
| M0 | 320 (74.1%) | 482 (85.2%) |
| M1 | 60 (13.9%) | 61 (10.8%) |
| unknown | 52 (12.0%) | 23 (4.1%) |
