# Supplementary material for: Establishment and Validation of a Genetic Label Associated With M2 Macrophage Infiltration to Predict Survival in Patients With Colon Cancer and to Assist in Immunotherapy
Source: Front Genet. 2021 Sep 6;12:726387. doi: 10.3389/fgene.2021.726387 (PMC8451970; doi:10.3389/fgene.2021.726387)
Supplement: Supplementary file 5 [file Table_2.DOCX]

Supplementary Material

**Table S2 The top 20 nodes in the gene co-expression network of tan module.**

| **Rank** | **Gene** |
| --- | --- |
| 1 | SPI1 |
| 2 | CD14 |
| 3 | LSP1 |
| 4 | ANKS4B |
| 4 | CD53 |
| 6 | CYBB |
| 7 | PLEKHO2 |
| 8 | PLEK |
| 9 | C1QA |
| 10 | FCGR3A |
| 10 | ITGB2 |
| 10 | TIMP1 |
| 13  13  13  13  13  18  18  20 | C1QB  CTSD  NFKB2  TREM2  TYROBP  GPNMB  ZNF703  ICAM1 |
